# STRONG for Surgery & Strong for Life — against all odds: intensive prehabilitation including smoking, nutrition, alcohol and physical activity for risk reduction in cancer surgery — a protocol for an RCT with nested interview study (STRONG-Cancer)

**DOI:** 10.1186/s13063-022-06272-2

**Published:** 2022-04-21

**Authors:** Hanne Tønnesen, Line Noes Lydom, Ulla Nordström Joensen, Ingrid Egerod, Helle Pappot, Susanne Vahr Lauridsen

**Affiliations:** 1 Clinical Health Promotion Centre, The Parker Institute, Bispebjerg-Frederiksberg Hospital, Part of Copenhagen University Hospitals, 2000 Frederiksberg, Denmark; 2 Department of Urology 2112, Copenhagen University Hospital, Rigshospitalet, 2100 Copenhagen, Denmark; 3 Department of Intensive Care 4131, Copenhagen University Hospital, Rigshospitalet, Copenhagen, Denmark; 4 Department of Oncology 5073, Copenhagen University Hospital, Rigshospitalet, 2100 Copenhagen, Denmark

**Keywords:** Perioperative risk reduction, Smoking, Alcohol drinking, Overweight, Obesity, Malnutrition, Physical activity, Bladder cancer, Radical cystectomy, Prehabilitation

## Abstract

**Background:**

There is a large unused potential for risk reduction in the preoperative period via effective lifestyle intervention targeting co-existing risky lifestyles: Smoking, malNutrition, obesity, risky Alcohol intake and insufficient Physical activity (SNAP).

This trial compares the efficacy of the integrated STRONG programme with standard care on preoperative risk reduction and secondly on SNAP factor improvement and frailty, postoperative complications and quality of life. A nested interview study explores the patient preferences and the multi-perspective view of patients, relatives and health professionals.

**Methods:**

In total, 42 surgical patients with ≥1 SNAP factor are allocated to individually tailored STRONG programme or usual care during adjuvant chemotherapy prior to radical bladder cancer surgery. The STRONG programme has ≥6 weekly sessions with patient education, motivational and pharmaceutical support. It is based on intensive smoking and alcohol cessation interventions reporting perioperative quit rates > 50%.

Surgical risk reduction is measured as ≥1 step for 1 or more risky lifestyles on the ASA-score, secondly as having no risky SNAP factors, and as any SNAP improvement. The outcomes are validated by measurements and biomarkers. Postoperative complications are categorised according to the Clavien-Dindo classification. Health-related quality of life is measured by EQ-5D.

The patients are followed up after 6 weeks at surgery and 6 weeks and 6 months postoperatively.

A representative sample of the participants, their relatives and the clinical staff are interviewed until data saturation. Transcription, triangulated analyses and data management are conducted using NVivo computer software.

**Discussion:**

The surgical agenda is characterised by fixed dates for surgery focusing on clear risk reduction within a short time. This requires a clinical useful lifestyle intervention programme with a high effect and coverage as well as containing all SNAP factors and tailored to individual needs.

The STRONG programme seems to meet these requirements. After development in multi-professional collaboration, STRONG is delivered by a specially trained nurse as part of the surgical patient journey.

Overall, this study will bring important new knowledge about risk reduction in a frail patient group undergoing major cancer surgery.

**Trial registration:**

Registration at www.clintrials.gov (NCT04088968)

The manuscript form from https://trialsjournal.biomedcentral.com/bmc/journal and the SPIRIT guidelines are followed.

## Administrative information

Note: the numbers in curly brackets in this protocol refer to SPIRIT checklist item numbers. The order of the items has been modified to group similar items (see http://www.equator-network.org/reporting-guidelines/spirit-2013-statement-defining-standard-protocol-items-for-clinical-trials/).

**Table:** 

Title {1}	STRONG for Surgery & Strong for Life - against all odds: Intensive Prehabilitation including smoking, nutrition, alcohol and physical activity for risk reduction in cancer surgery – A protocol for an RCT with nested interview study (STRONG-Cancer)
Trial registration {2a and 2b}.	Registration at www.clintrials.gov (NCT04088968) The manuscript form from https://trialsjournal.biomedcentral.com/bmc/journal and the SPIRIT guidelines are followed.
Protocol version {3}	2021 – August 28
Funding {4}	The Danish Cancer Society (R223-A13094): 2.9 mill. DKK (part of the COMPAS Project receiving 20 mill. DKK); Novo Nordic Foundation (NNF19OC0058924): 0.6 mio DKK; the Parker Institute, Bispebjerg-Frederiksberg Hospital is supported by a core grant from the Oak Foundation (OCAY-18-774-OFIL).
Author details {5a}	1 Clinical Health Promotion Centre, The Parker Institute, Bispebjerg-Frederiksberg Hospital, Part of Copenhagen University Hospitals, 2000 Frederiksberg 2 Department of Urology 2112, Copenhagen University Hospital, Rigshospitalet, 2100, Copenhagen 3 Department of Intensive Care 4131, Copenhagen University Hospital, Rigshospitalet, Copenhagen, Denmark 4 Department of Oncology 5073, Copenhagen University Hospital, Rigshospitalet, 2100, Copenhagen
Name and contact information for the trial sponsor {5b}	hanne.tonnesen@regionh.dk (ORCID ID: 0000-0002-7161-3416)
Role of sponsor {5c}	The funders play no role in the conceptualism, the design, collection, management, analyses and interpretation of data, writing the report for publications or have ultimate authority over any of these activities.

## Introduction

### Background and rationale {6a}

#### The problem to be solved

Surgery remains the gold standard of treatment for many diseases, but the outcomes are poor among frail patients with unhealthy lifestyles; thus, strategies to optimise the patient group are increasingly important [1–7]. The improved intraoperative techniques and enhanced recovery after surgery (ERAS), e.g. the ERAS protocol, are important [8]. However, there is a large unused potential of risk reduction in the preoperative period [9], in particular the modifiable risk factors, which can be targeted with preventive strategies [10].

Even in case of screening for smoking, nutrition, alcohol and physical activity (SNAP) at surgery, screening results are only fragmentally followed up by systematic interventions in the clinical pathways for patients with cancer and other diseases [11]. This is despite the World Health Organization (WHO) recommendations and the surgical guidelines endorsing all patients to be offered evidence-based support for risk reduction. In 2014, the American Society for Anesthesiologists (ASA) included relevant details on alcohol, smoking and obesity in the international preoperative risk evaluation for adults, the ASA-score [12].

The SNAP factors often co-exist and each add to the surgical risk [13–15]: about 50% for daily smoking, alcohol > 2 drinks/day [16] and severe malnutrition — similar to the risks related to severe cardiac, pulmonary and kidney insufficiency. Obesity even at cancer surgery [17–19] and low physical activity are followed by 10–20% increase [20]. Frailty is an example of co-existing SNAP factors with a major impact on the surgical outcome [21, 22].

#### Pathophysiological mechanisms

Patients with an unhealthy lifestyle develop the same type of complications as all other patients, just more frequently [23–26]^.^ The pathophysiology involves a comprehensive suppression of several organ systems of importance for surgical outcomes. They dysfunction prior to surgery and add to the surgical trauma itself, targeting the same organ functions. For alcohol, the preoperative organ dysfunction even exists in patients without, e.g. alcohol-induced liver disease, and for smoking without, e.g. smoking-induced lung disease [27]. The organ damage is often subclinical and takes place at the cellular level, thus reducing the extra capacity that usually supports the patient successfully during surgery and recovery.

#### Need for a combined programme

The risk at surgery increases with co-existing risk factors, but no integrated programme exists targeting all SNAP factors [5, 20, 28]. We have shown that an intensive intervention aiming at complete smoking or alcohol abstinence for 4 to 8 weeks halves the postoperative complication rate and indicates a sustained effect for longer time [29, 30]. Preoperative inspiratory exercise for 1–2 weeks reduces postoperative pneumonia [31], while malnutrition intervention before surgery is part of the international guidelines [6, 7, 32]. General physical exercise reduces the postoperative recovery, but the impact on complications is disappointing [20, 33, 34]. Preoperative obesity and frailty interventions are still sparsely investigated in cancer surgery [18, 35–37].

Recently, our group successfully tested a combined smoking and alcohol cessation intervention [38]. We also identified a minimal impact of the social gradient after a successful intensive smoking cessation intervention [39]. In addition, the social inequality in health promotion success could be minimised through positive/selective involving procedures and support during the total intervention [40]. Our STRONG programme will build on these results.

#### The frameworks

**Fig. 1:**
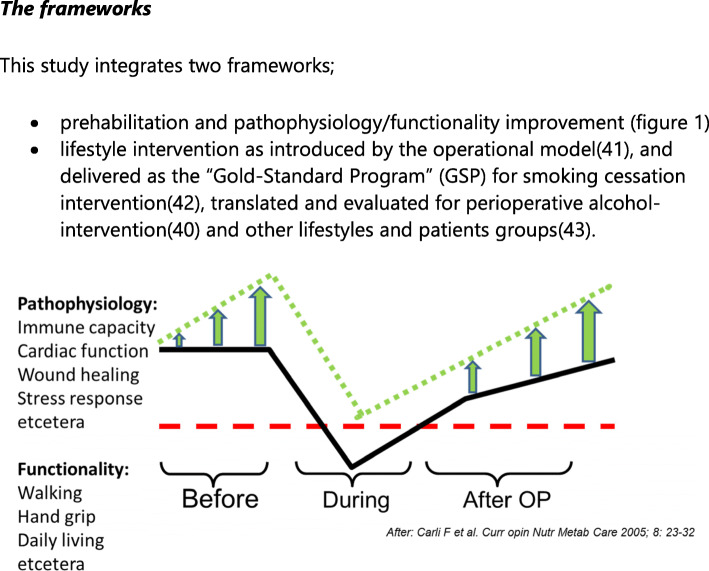
Potential of preoperative improvement by intensive SNAP prehabilitation

This study integrates two frameworks:

- Prehabilitation and pathophysiology/functionality improvement (Fig. 1)
- Lifestyle intervention as introduced by the operational model [41], and delivered as the “Gold-Standard Program” (GSP) for smoking cessation intervention [42], translated and evaluated for perioperative alcohol intervention [40] and other lifestyles and patient groups [43]

#### Preference among patients, relatives and staff

Cancer diagnosis and surgery are considered “windows of opportunity” for a successful lifestyle change. A common staff barrier (or misconception) is “that it is unwanted by the patient” which might imply judgements. Interestingly, the patients often express a need for being involved in their own recovery, regarding perioperative lifestyle intervention as part of the surgical treatment [44, 45]. By nesting interviews of patients, relatives and staff, we will provide triangulated perspectives of preferences, and experienced facilitators and barriers for the STRONG programme.

Overall, it is necessary to develop a combined programme with a strong potential to reduce the high risk at surgery, originating from all the SNAP factors. Our STRONG programme builds on the existing evidence, including the feasibility to be delivered by specially trained nurses in the complex surgical setting.

### Objectives {7}

The main objective is to compare the efficacy of the intensive prehabilitation STRONG aiming at risk reduction with standard care for patients with at least one SNAP factor (Smoking, Nutrition – obesity and/or malnutrition - risky Alcohol drinking and low Physical activity) prior to bladder cancer surgery and secondly to compare the improvement of SNAP factors, with frailty, postoperative complications and health-related quality of life. Furthermore, we aim to explore the patient preferences as well as a multi-perspective view of the STRONG programme.

The main hypothesis is that intensive SNAP prehabilitation reduces the high surgical risk related to the five risky lifestyles compared to treatment as usual.

### Trial design {8}

This is a superiority intervention study in a randomised design with parallel groups using allocation 1:1. An interview study is nested in the trial.

## Methods: participants, interventions and outcomes

### Study setting {9}

**Fig. 2:**
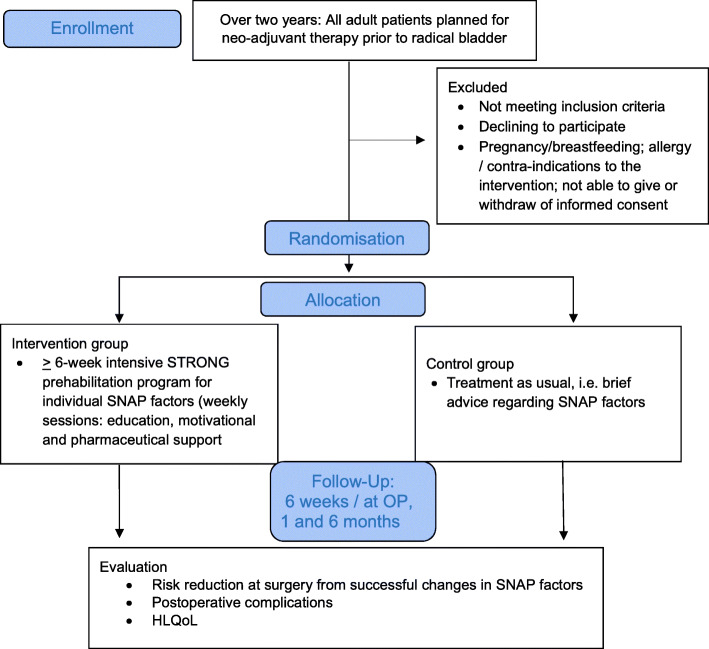
Trial profile

The patients are recruited from the Department of Urology, and the intervention takes place during their preoperative chemotherapeutic treatment at the Department of Oncology, both at Copenhagen University Hospital, Rigshospitalet, Copenhagen. The intervention is performed by the surgical STRONG team anchored at the Clinical Health Promotion Centre, The Parker Institute, Bispebjerg-Frederiksberg Hospital, Part of Copenhagen University Hospitals, Denmark. A panel of patients with personal experience of cancer surgery is formed and actively involved in the research process (Fig. 2).

### Eligibility criteria {10}

Inclusion criteria: Patients > 18 years, at least one of the five SNAP factors (defined below) and scheduled for radical cystectomy (RC) due to bladder cancer and referral to neo-adjuvant chemotherapy preoperatively

- “Smoking”: daily smoking — no limitations [29]; validated by carbon monoxide (CO) in the breath test
- “Obesity”: BMI above 30 [46], validated by weight and height
- “Malnutrition”: based on ESPEN guidelines for surgery including albumin concentration in blood [6]
- “Risky drinking”: > 2 standard drinks per day (in average; 1 drink = 12 g ethanol), identified by timeline follow-back (TLFB) [47], and validated by alcohol biomarkers (carbohydrate-deficient transferrin (CDT), plasma phosphatidylethanol (P-Peth) and urine ethylglucuronide (U-EtG))
- “Physical inactivity”: < 30 min physical activity per day or 3.5 h per week

Exclusion criteria: pregnancy and breastfeeding as well as allergy and other contra-indications to pharmaceutical support, exercise or nutritional intervention; inability to give informed consent (e.g. low age, severe mental illness including delirious conditions, consciousness issues, local language challenges and similar) and withdrawal of informed consent.

### Who will take informed consent? {26a}

In connection with the initial planning of surgery, the patient’s designated urologist and a clinical project nurse approach eligible patients and invite them to participate in the study.

A project nurse will give oral and written information and answer questions. The informed consent is collected as soon as possible to allow for at least 6 weeks of prehabilitation during the preoperative oncological treatment period (about 8–12 weeks).

### Additional consent provisions for collection and use of participant data and biological specimens {26b}

During the project period, new markers may be developed, and we ask participants separately about giving informed consent for future studies.

## Interventions

### Explanation for the choice of comparators {6b}

For comparison, a control group is necessary in this study, as this is the first RCT to evaluate an intensive lifestyle intervention including up to five SNAP factors. Though theoretically possible from the literature as presented above, we do not know if and to which degree it is possible for the patients to successfully change several risky lifestyles at the same time during the perioperative period.

The patients undergoing major bladder cancer surgery are chosen because they may benefit very much from a perioperative lifestyle intervention. Smoking induces urothelial cancer, and the candidates for radical cystectomy constitute a group with a short-term high perioperative risk and a long-term health reduction. They are characterized by age around 70 years, a high comorbidity [5], about 25% at severe nutritional risk and 30% current smokers. Still, obesity is seldom seen in patients undergoing RC [48] but a one-unit increase in body mass index (BMI) has been significantly associated with a higher risk of a major complication in patients undergoing RC [19].

Overall, these patients are considered a frail population with complication rates up to 64% within 3 months, postoperatively [49, 50].

### Intervention description {11a}

The intervention group receives the intensive STRONG prehabilitation programme tailored to meet the individual patient’s need for risk reduction at surgery. It has minimum five educational sessions over 6 weeks (i.e. about one weekly), motivational support and pharmaceutical support such as nicotine replacement therapy as indicated. The education relates directly to their surgical treatment and includes introduction to STRONG, motivational level, ambivalence, pros and cons; symptoms of addiction and/or withdrawal (experience and expectations); relapse (description and management); benefits of short- and long-term lifestyle change; continued lifestyle change following STRONG, continued education based on the current conditions.

STRONG is introduced with the surgical recommendations in ‘Engage in the process of change’ [41]. The smoking follows the Gold Standard Programme (GSP) [42], which also has been translated to the standardised alcohol cessation intervention [40], exercise training programme and nutritional intervention [43].

The STRONG programme focuses on healthy days, defined as days without smoking or drinking, eating according to the nutritional plan and being physically active for at least 30 min. The number of SNAP factors involved relates to the individual patient’s unhealthy lifestyles. The content of the programme is recommended based on symptoms and chosen by the patients from a roll-down menu, e.g. type of nicotine replacement therapy (NRT), alcohol withdrawal prophylaxis, combination of food or nutritional supplement adding up to the intake needed and type of physical activity.

The control group also receives the participant information and standard care, which includes that smokers are offered a very brief advice (VBA) and referral to a municipal clinic for smoking cessation intervention.

All participants receive the national folders on smoking and alcohol advise in relation to surgery. All are free to use any support offered outside the study, including the free access to lifestyle intervention in their municipality. Patients are asked if they have experienced side effects, particularly of the pharmacological support. Potentially unknown side effects are reported and, if serious, may lead to early termination of the trial.

Clinical project nurses, who have taken part in a 5-day educational programme followed by practical training in the STRONG programme based on the GSP, provide the intervention. The daily project leader (SVL) ensures that the counsellor follows the principles of the GSP programme by discussion of the intervention regularly.

### Criteria for discontinuing or modifying allocated interventions {11b}

A participant may withdraw from the study at any time without this impacting on any future investigations and/or treatment at the site, by the project team or other staff associated with the study. The project team may discontinue any participant’s participation, e.g. due to an adverse event and safety concerns. The principal investigator has the right to terminate the study. Reasons may include unsatisfactory fulfilment of the design, participant enrolment, time schedule and administrative agreements.

### Strategies to improve adherence to interventions {11c}

The adherence is measured as meeting adherence during the intervention and follow-ups. In addition, the lifestyle changes are monitored by interviews and validated by markers.

### Relevant concomitant care permitted or prohibited during the trial {11d}

All participants are free to use any support offered outside the project, including the free access to lifestyle intervention in their municipality.

### Provisions for post-trial care {30}

In case of needs (for post-trial care and others), the participants are followed long-term at the Department of Urology and/or Department of Oncology.

### Outcomes {12}

#### Risk reduction at surgery from changes in lifestyle

Primary outcome: Number of patients with surgical risk reduction of at least 1 step for 1 or more risky lifestyles on the ASA-score [51] after 6 weeks which is the end of the intervention.

Secondly: Number of patients without their preoperative risky lifestyles at 6 weeks after inclusion, at surgery and at 6-week and 6-month follow-up postoperatively:

- Successful smoking cessation, validated by CO in the breath test and U-cotinine
- Malnutrition: Not at risk of malnutrition (ESPEN surgical guidelines, incl. albumin concentration) [6]
- Obesity: body mass index (BMI) < 30 or at least 5% loss of body fat mass and max 10% for obesity at 6 weeks (0.5–1 kg/weekly and below 1% gain of body fat mass at 6 months (without developing malnutrition) [52]
- Successful alcohol cessation at 6 weeks and intake below risky limits at 6 months, validated by CDT, P-Peth and U-EtG
- Physical activity at least 30 min per day or 3.5 h per week and 6-min walk test (6MWT) > 500 m

Number of patients with any reduction of any of their lifestyles, i.e. reduced number of cigarettes, amount of alcohol intake and body fat mass, level of malnutrition as well as increased body-muscle mass, minutes of physical activity per day and 6MWT. Number of patients with reduced frailty level, measured by Lammers definition [22].

##### Health-related quality of life

Measured by EQ-5D [53] with 5 dimensions and a visual analogue scale (VAS). This generic quality of life instrument used in clinical studies reflects the severity of five dimensions, mobility, self-care, usual activities, pain/discomfort and anxiety/depression, at three levels of severity. Each of the five dimensions is divided in five levels of perceived problems. The higher level, the more problems are experienced. Overall health is measured with a visual analog scale (VAS) from 0 to 100 with a higher score representing a better health. The questionnaire is cognitively undemanding, and it takes only a few minutes to complete. It has been found well-functioning in older age groups comparable with the population in this study protocol.

#### Postoperative complications

Postoperative complications are defined by requiring documented treatment within 30 days and 6 months after surgery, categorised in accordance with the Clavien-Dindo grading [54] as well as the Comprehensive Complication Index (CCI) [55]. They include adverse events and side effects (Table 1).

**Table 1:** Outcome collection during the study

Outcomes	Week 6 (FU)	Surgery	Follow-up 30 days	Follow-up 3 + 6 months
Main: Risk reduction of at least 1 step for at least 1 risky lifestyle (ASA-definition)				
Yes/no	X	X	X	X
Level (going from–to)	X	X	X	X
Secondary outcomes				
Quitting the risky lifestyles (as intervened)	X	X	X	X
Any reduction in lifestyles or frailty	X	X	X	X
Hospital Anxiety and Depression Score (1Q)	X	X	X	X
General HR-Quality of life (EQ-5D)	X	X	X	X
Complications (yes/no)			X	X
Clavien-Dindo (grade) and CCI			X	X

### Participant timeline {13}

The patients are enrolled, allocated and begin the intervention about the same date, which is at least 6 weeks prior to surgery.

The semi-structured interviews take place after ending the STRONG programme using an interview guide developed by the study team, based on the study aim, previous prehabilitation research [56] and concepts in the frameworks described above.

### Sample size {14}

Overall, we will include 42 patients. The power calculation regarding the main outcome is based on meta-analyses of quit rates after about 6 weeks at 70% (ranging 50–90%) [29, 30] after the intensive interventions like GSP for smoking and alcohol cessation intervention in the perioperative period as well as on studies reporting completion of physical exercise programmes at 66%, while the effect in the control groups were 15% (5–25%) [33, 57]. The corresponding lifestyle-related risk reduction will go from 100 to 30% in the intervention group and from 100 to 85% in the control group. Using an 80% power and 2 × alpha = 0.05, this would result in enrolment of 2 × 11 patients. However, the effect of obesity intervention is not known in patients with cancer undergoing major bladder surgery, but the number of patients with obesity seems low based on clinical experience; thus, a conservatively estimated sample size based on a lower effect of the intervention and allowing for dropouts would be 2 × 21 patients.

The number of participants in the interviews is determined by the concept of information power which will guide the adequate sample size. This entails an ongoing reflection during the study considering issues such as the aim of the study, sample specificity, use of a theoretical background and the chosen analytical strategy [58]. In this study, we propose to include about 8 patients from the RCT study and their relatives and clinical staff.

### Recruitment {15}

The patient flow at the department will ensure adequate enrolment to reach the target sample. All patients are evaluated for eligibility in the study period. Recruitment, intervention and follow-up take place during the daytime on working days and are planned together with the participants. The interviews can be conducted at the hospital or at home according to the wishes of the participant. From previous studies on the patient group, we have experienced that only few participants leave the lifestyle intervention trials [33, 48].

## Assignment of interventions: allocation

### Sequence generation {16abc}

After informed consent, the participants will be randomised by the project nurse to the intensive lifestyle intervention or standard care using a computer-generated block randomisation scheme [59]. The computerised randomisation system is accessible around the clock, and the use is logged. This ensures immediate randomisation of patients accepting participation and adequate allocation concealment. Block sizes vary from 2 to 5.

## Assignment of interventions: blinding

### Who will be blinded {17a}

It is rarely possible to blind intensive lifestyle intervention. The project data are kept away from the medical record system, though it cannot be guaranteed that the participant does not spontaneously tell the clinicians and the outcome assessor about the intervention group allocation. However, all biomarker analyses for validation and the statistical analyses will be performed blinded. Likewise, the measurement of postoperative complications is done by a blinded urologist not involved in the project.

### Procedure for unblinding if needed {17b}

The design is open label with outcome assessors and data analysts being blinded so unblinding will not occur.

## Data collection and management

### Plans for assessment and collection of outcomes {18a}

Project staff will collect data; please see Tables 2 and 3. The data for analyses are performed blinded and there will be no personal identification in the presentation of results. Complications will be registered using predefined definitions by two assessors blinded to the allocation group.

**Table 2:**
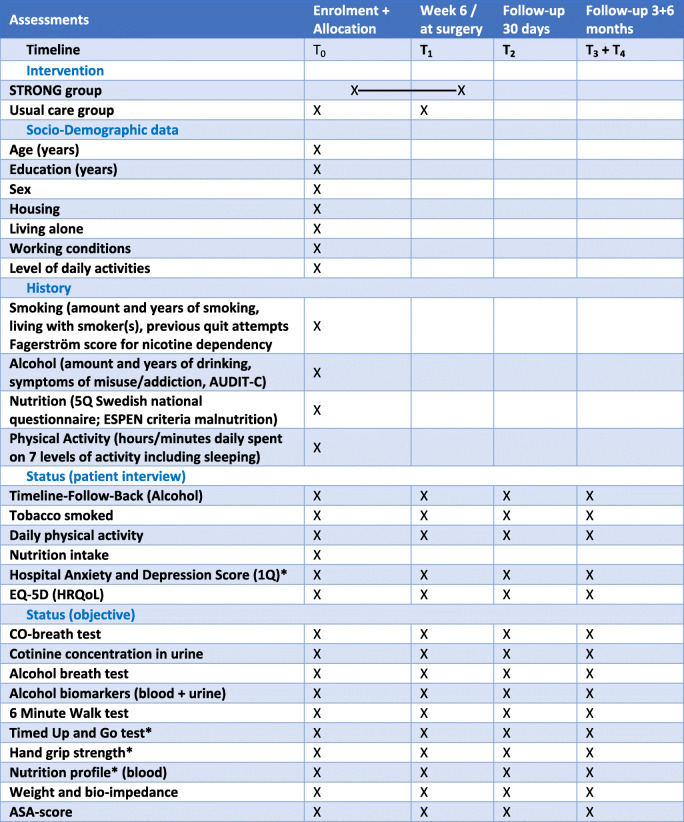
Schedule of enrolment, interventions and assessments for all patients (*included in the frailty definition)

**Table 3:** Further weekly assessments in the STRONG intervention group

Assessments (STRONG group)	Meet 1	Meet 2	Meet 3	Meet 4	Meet 5	cont. until surgery
Smoking						
Cigarettes per day (N^o^)	X	X	X	X	X	X
NRT (mg) as craving reducing medicine	X	X	X	X	X	X
Validation by CO concentration (breath)	X	X	X	X	X	X
Alcohol						
Alcohol per week (units 12 g, TLFB)	X	X	X	X	X	X
Alcohol concentration (breath test)	X	X	X	X	X	X
Alcohol biomarkers (blood + urine)	X	X	X	X	X	X
Withdrawal symptoms (CIWA-R)	X	X	X	X	X	X
Use of AWS prophylaxis (mg)	X	X	X	X	X	X
Nutrition						
Food intake according to plan	X	X	X	X	X	X
Appetite level /healthy days	X	X	X	X	X	X
Weight and bio-impedance	X	X	X	X	X	X
Validation by nutrition profile in blood	X	X	X	X	X	X
Physical activity						
Physical activity according to plan	X	X	X	X	X	X
Steps walked per day	X	X	X	X	X	X
Validation by 6-min walk test	X	X	X	X	X	X
Timed Up and Go test	X	X	X	X	X	X
Hand grip strength	X	X	X	X	X	X

### Plans to promote participant retention and complete follow-up {18b}

All meetings are planned to take place together with the usual contacts in the patient journey. In case of dropout, we will follow our previous procedure and ask the patient for written consent to allow follow-up in the medical record system. This is included in the application for the Scientific Ethical Committee.

### Data management and confidentiality {19,27}

The data are collected and securely stored in the Redcap system using a project ID for each participant instead of personal identifiable information, as recommended by the Capital Region of Denmark, where the trial takes place. The key between the project ID and the personal identification is stored under a double lock. Only the project group have access to the data.

The conduction of the project follows the Danish Data Protection Agency guidelines of the European GDPR before, during and after the trial. After ending the trial, the personal information is destroyed and the other data are stored at the Danish National Data Archive — also fulfilling the Danish and European guidelines for data management and protection.

### Plans for collection, laboratory evaluation and storage of biological specimens for genetic or molecular analysis in this trial/future use {33}

In this study, all patients have a blood test for the identification of alcohol and/or smoking biomarker and for nutrition profile analyses (2 × 5 ml whole blood and 2 × 2 ml EDTA plasma, 2 × 2 ml citrate plasma, 2 × 2 ml serum, 5 × 2 ml urine): B-haemoglobin, B-MCV, P-gamma GT, P-Asat, P-bilirubin, p-albumin and p-protein. Of those, blood (10 ml) and urine (5 × 2 ml) will be sampled and stored for later identification of alcohol biomarkers (PetH, CDT and U-EtG).

The aim is to validate the self-reported data on lifestyle outcomes. The analyses of biomarkers are performed together for one factor at a time, mainly after collection of all samples. Therefore, it is necessary to collect and store the samples during the project period at the Parker Institute, Bispebjerg-Frederiksberg Hospital. The samples will be destroyed before 31.12.2030.

All analyses are expected to be performed locally at the Parker Institute, Bispebjerg-Frederiksberg.

The data handling is performed in agreement with GDPR, and the project is registered at the Data Protection Agency before start.

During the project period, new markers may be developed, and we would therefore keep half of the samples for future analyses of biomarkers related to measurement of markers for risk reduction at surgery in the future. The participants are separately asked about giving informed consent for that, understanding that a future measurement and data handling is performed in agreement with GDPR.

## Statistical methods

### Statistical methods for primary and secondary outcomes {20a,b,c}

The effect of the intervention on risk reduction at surgery and SNAP improvement is analysed by Fisher’s exact test on an intention-to-treat basis. Estimates of the difference between treatment groups will be reported as relative risks (RR) with 95% confidence intervals (CI) and *p* values. Effects of the intervention Health-Related Quality of Life scores are analysed by Mann-Whitney’s *U* test. *p* values below 0.05 and CI not including the value 1 are considered significant. The analyses are done by Stata®.

For the interviews, the transcription, the triangulated analyses and data management will be conducted using NVivo QSR International.

### Interim analyses {21b}

No interim analyses will be performed.

### Methods for additional analyses (e.g. subgroup analyses) {20b}

No subgroup analyses are planned.

### Plans to give access to the full protocol, participant-level data and statistical code {31c}

The datasets analysed during the current study and statistical code will be available from the corresponding author on reasonable request, as well as the full protocol.

## Oversight and monitoring

### Composition of the coordinating centre and trial steering committee {5d}

The project is run day-to-day and coordinated by in a collaboration between the three involved departments: the STRONG team, the Department of Urology and Department of Oncology.

The patient panel meets twice annually.

The trial steering committee includes the principal investigators from the 7 studies under the COMPAS project holding the funding. They meet 4 times a year.

### Composition of the data monitoring committee, its role and reporting structure {21a}

The data monitoring is done by the STRONG team on a daily basis and controlled quarterly by an external independent researcher.

### Adverse event reporting and harms {22}

Information of adverse events to the interventions is collected at every meeting and if any occur they will be reported to the Danish Medicines Agency. In addition, the participants can also contact the staff via the hotline.

### Frequency and plans for auditing trial conduct {23}

The project management group meets once a month. The Trial Steering Group and the monitor group have their meetings quarterly. In case of needs, extra meetings will take place. The Danish Scientific Ethical Committee and the Danish Protection Agency use unannounced visits for reviewing.

### Plans for communicating important protocol amendments to relevant parties (e.g. trial participants, ethical committees) {25}

The study has been approved by the Danish Scientific Ethical Committee (H-20081571) and by the Danish Protection Agency (P-2020-95). We will communicate important protocol modifications to the involved parties, as well as to the Danish Scientific Ethical Committee, Danish Protection Agency, and update trial registries, including www.clinicaltrials.gov as soon as possible.

### Dissemination plans {31a}

All results will be published — positive, negative or inconclusive, including at www.clinicaltrials.gov, when the study is finalised. The results will be disseminated in scientific journals, and authorships follow the Vancouver Criteria. Furthermore, the results will be spread to the public at the website, by lecturers and through clinical, research and public networks.

## Discussion

This study evaluates the risk reduction effect of combined intensive interventions for up to five unhealthy lifestyles in the preoperative period during chemotherapeutic treatment prior to radical bladder resection for urothelial cancer.

From a public health perspective, there is an effect of even brief and even very brief lifestyle intervention, which is therefore often recommended [60]. However, this effect is relatively small, though measurable and cost-effective at the population level and therefore important on the longer term.

By contrast, the surgical agenda characterised by fixed dates for surgery and focus on clear risk reduction within short time, thus moving the high-risk patient to a lower risk level for complications and faster recovery — already before the operation.

Therefore, the STRONG programme uses the highly effective intensive intervention, the GSP [42], that has proven significant quit rates of about 50% for smoking and risky alcohol intake at short term followed by a similar reduction of clinical complications in surgical settings [61]. They may also be followed by a long-term effect as well [29]. Unfortunately, the briefer interventions have a lower effect on lifestyles and until now no effect on complications [29].

In addition to the intensity of the intervention, also the aim of complete lifestyle change is of importance to receive a clear risk reduction prior to surgery. Reducing, but not quitting smoking or risky alcohol intake, does not significantly impact the complication rates [61, 62].

From a clinical perspective, it is relevant to get the most surgical risk reduction from an intervention and therefore put the focus on the unhealthy lifestyles followed by the highest risk, i.e. daily smoking, alcohol intake exceeding 2 drinks per day or 14 per week and malnutrition defined by the ESPEN surgical guidelines. However, when looking into co-existing risk factors, there seems to be an interaction on the risk, as shown for smoking and increasing BMI [63]. Therefore, it makes sense to evaluate the effect of the combined STRONG for all the five risk factors, in order to harvest the unused potential for risk reduction.

The STRONG programme has been developed in multi-professional collaboration, but it is intended to be feasible for delivery for individual patients by an especially trained nurse as part of the already existing patient journey in relation to surgery.

This approach is based on our experiences hitherto regarding perioperative smoking and alcohol intervention, the patient preferences and the applicability in daily surgical life, if the study shows an effect on lifestyles.

In another patient group in treatment for alcohol and drug addiction, we have pilot tested a combined programme involving a nurse-led smoking cessation intervention, a dietician-led nutrition counselling, a physiotherapist-led training programme and a physician-led participant education programme [64, 65]. In practice, it was not possible for the participants with several risky lifestyles to follow all the individual interventions, which also required additional administrative resources to manage. However, the most interesting experience was that the participants had similar considerations regarding pros and cons for changing the different lifestyles, or in other words, they saw themselves as one integrated person. Therefore, we re-arranged the intervention to fit to individual patients by containing all relevant lifestyles at the same session — and trained the nurses to deliver that — instead of trying to make the patients fit into the fragmented intervention [43]. This combined approach has also been used in our most recent intervention trial on perioperative smoking and alcohol intervention, which was welcomed by the patients [44].

Overall, this study will bring important new knowledge into the clinical research field of risk reduction in a frail patient group undergoing major cancer surgery. If it is possible to significantly reduce the increased surgical risk from unhealthy lifestyles and identify related perspectives from the patients, relatives and staff by the STRONG programme, we will get a new platform for preoperative risk reduction through a combined lifestyle intervention. The results would then be integrated in further research on postoperative complications, pathophysiology and long-term health gain. If these high-risk patients do not benefit from the intervention, new strategies are required.

## Trial status

Trial status: Opens for inclusion in Autumn 2022. And we expect recruitment to be completed within 2 years.

Protocol version number 2 (13.01.2022).

## Abbreviations

ASA: American Society for Anesthesiologists
AUDIT-C: Alcohol Use Disorders Identification Test
BMI: Body mass index
CCI: Charlsons comorbidity index
CDT: Carbohydrate-deficient transferrin
CI: Confidence interval
CO: Carbon monoxide
EQ-5D: EuroQual-5D — a standardised instrument for use as a measure of health outcome
GSP: Gold Standard Programme
HRQoL: Health-related quality of life
NRT: Nicotine replacement therapy
PEth: Phosphatidylethanol
RC: Radical cystectomy
RR: Relative risk
SNAP: Smoking, nutrition (obesity and malnutrition), alcohol, physical activity
VAS: Visual analogue scale
